# Detecting Pest-Infested Forest Damage through Multispectral Satellite Imagery and Improved UNet++

**DOI:** 10.3390/s22197440

**Published:** 2022-09-30

**Authors:** Jingzong Zhang, Shijie Cong, Gen Zhang, Yongjun Ma, Yi Zhang, Jianping Huang

**Affiliations:** School of Mechanical and Electrical Engineering, Northeast Forestry University, Harbin 150040, China

**Keywords:** deep learning, attention mechanism, Sentinel-2, semantic segmentation, pest area detecting, vegetation indices

## Abstract

Plant pests are the primary biological threats to agricultural and forestry production as well as forest ecosystem. Monitoring forest-pest damage via satellite images is crucial for the development of prevention and control strategies. Previous studies utilizing deep learning to monitor pest-infested damage in satellite imagery adopted RGB images, while multispectral imagery and vegetation indices were not used. Multispectral images and vegetation indices contain a wealth of useful information for detecting plant health, which can improve the precision of pest damage detection. The aim of the study is to further improve forest-pest infestation area segmentation by combining multispectral, vegetation indices and RGB information into deep learning. We also propose a new image segmentation method based on UNet++ with attention mechanism module for detecting forest damage induced by bark beetle and aspen leaf miner in Sentinel-2 images. The ResNeSt101 is used as the feature extraction backbone, and the attention mechanism scSE module is introduced in the decoding phase for improving the image segmentation results. We used Sentinel-2 imagery to produce a dataset based on forest health damage data gathered by the Ministry of Forests, Lands, Natural Resource Operations and Rural Development (FLNRORD) in British Columbia (BC), Canada, during aerial overview surveys (AOS) in 2020. The dataset contains the 11 original Sentinel-2 bands and 13 vegetation indices. The experimental results confirmed that the significance of vegetation indices and multispectral data in enhancing the segmentation effect. The results demonstrated that the proposed method exhibits better segmentation quality and more accurate quantitative indices with overall accuracy of 85.11%, in comparison with the state-of-the-art pest area segmentation methods.

## 1. Introduction

Forests play a crucial part in preserving the natural environment’s biodiversity as well as the cyclical flow of energy and mineral elements in the biosphere, which are essential to the ecosystem. The effect of outbreaks of forest pests on healthy forests is severe. Consequently, research on forest pests and their impacts has received considerable attention over the years [1]. It is essential to boost the management of forest pests to minimize the growth of diverse forest pests, improve forest quality, protect the genetic integrity of forest species, maintain ecological balance, and promote coordinated ecological development. The bark beetle, which is a species of the Coleoptera order, *Scolytidae* family, is among the most devastating pests in western North America [2]. It has a significant symptom lag and is highly insidious. In the last few decades, billions of trees have been destroyed by it in the US and Canada [3], causing severe damage to North American forests. The aspen leaf miner is a transcontinental pest of trembling or small tooth aspen, feeding on the poplar leaves’ epidermal cells [4]. It has wreaked havoc in the northwestern Canadian and Alaskan forests since the late 1990s, leading to a wide array of consequences [5,6]. Its epidemics can last for a decade or longer [7]. Both pests have a significant impact on timber production in North America and destabilize the ecological equilibrium of local forests. For the protection of forest ecosystems, their timely and accurate monitoring is crucial.

Traditional monitoring techniques rely on field surveys conducted by personnel. However, traditional manual monitoring methods are time-consuming, making it difficult to meet the application requirements for rapid pest monitoring during production. Field surveys are constrained by the natural environment, and many areas of the field are inaccessible to humans. Large-scale, objective, rapid, and non-destructive are all characteristics of remote sensing technology. For this reason, a growing number of researchers are employing remote sensing technology to monitor forest pests.

Unmanned aerial vehicles (UAVs) have emerged as valuable tools in the monitoring of forest health over large areas. Safonova, et al. [8] extracted areas of UAV imagery that might contain tree crowns and then assessed the degree to which they had been damaged by the Blandford bark beetle. Yu, et al. [9] utilized UAV to monitor pine wilt disease (PWD) in pine forests at an early stage. Many forest areas, however, are unsuitable for the use of UAVs for monitoring due to limitations imposed by sovereignty and natural conditions (e.g., uninhabited areas and uninhabited islands). Although UAVs have the capability to conduct detailed surveys of regional pests, it is difficult for them to conduct a large-scale pest census due to factors such as endurance and relatively limited detection scope.

Satellite remote sensing has a vast observation range, and the cost for the same monitoring area is less than that of UAVs. In addition, it can observe continuously for an extended period of time and generate time-series data. Unlike traditional monitoring techniques and UAV monitoring, satellite remote sensing makes it easier to obtain time-series data without requiring repeated surveys by traveling to the field at specified intervals. In recent years, numerous studies have demonstrated that satellite remote sensing can effectively evaluate the health of insect-infested forests. Dennison, et al. [10] classified mountain pine beetle-damaged canopy cover areas using panorama-sharpened GeoEye-1 images. Meddens and Hicke [3] used multi-temporal Landsat data to analyze and predict tree death due to mountain pine beetle. Using a support vector machine algorithm, GF-2 and Sentinel-2 imagery in combination for detecting individual and stand-scale tree mortality, respectively, that was attributed to red turpentine beetle, by Zhan, et al. [11]. With its multi-temporal, high-resolution, and large-scale range characteristics, satellite remote sensing technology has gradually become an essential instrument for forest-pest surveillance. Its application provides new opportunities for enhancing the location, nature, area range, and frequency assessments of forest pests.

The identification of forest-pest areas in satellite images is a classification task for remote sensing images. With the continuous development of deep learning technology in recent years, numerous researchers have applied it to the field of remote sensing imagery classification [12]. Hong, et al. [13] developed a general multimodal deep learning framework to fuse multimodal data for the classification of surface objects in remote sensing images. Wu, et al. [14] proposed a plug-and-play cross-channel reconstruction module, which enables more efficient information exchange between different remote sensing data and provides new insight into the task of land cover classification. A novel version of graph convolutional networks (GCNs) was proposed by Hong, et al. [15] that outperformed traditional GCNs for the task of hyperspectral remote sensing image classification. Shi, et al. [16] integrated spatial and spectral information in order to reduce information loss during feature extraction and increase the accuracy of land cover classification. The classification of remote sensing images can be solved by image semantic segmentation methods [17]. It is one of the core elements of computer vision research, which analyzes the content of the involved images using various algorithms, allowing the artificial intelligence system to recognize the images’ semantics at the pixel level. Image semantic segmentation can be used to extract the areas damaged by bark beetle and aspen leaf miner in satellite images, thereby classifying pest-affected areas in remote sensing images.

However, in this field, multispectral data and deep learning are not combined. RGB bands were utilized in prior studies that employed deep learning to identify pest areas in the forest. For instance, Kislov, Korznikov, Altman, Vozmishcheva, Krestov, Disney and Cord [18] segmented areas of damaged forest in RGB bands of Worldview-2 and Worldview-3 images using deep convolutional neural network. Using a deep learning approach, Zhou, et al. [19] identified trees damaged by PWD in BJ-2 images (RGB). Multispectral data contain more spectral information than RGB data. Insect damage, unlike physical damage such as storms and floods, can significantly alter the internal chemical composition of trees [20]. For analyses involving the chemical composition of plant tissues, extensive spectral information is essential [21,22,23]. Using vegetation indices generated from multispectral data, some researchers have obtained favorable results in pest monitoring [24,25,26,27,28,29]. Bárta, et al. [30] employed a random forest algorithm to analyze seasonal changes in vegetation indices of Norway spruce forests in Sentinel-2 imagery in order to monitor bark beetle infestations, achieving an overall accuracy of 78% in separating healthy and green attack categories. Choi, et al. [31] used Landsat image-based computation of NDMI to detect defoliation caused by the Asian gypsy moth. Kern, et al. [32] used NDVI calculated from MODIS data to detect oak lace bug hazard in oak forests and compared it to field data with greater than 61.1% agreement. Using Fisher discriminant analysis, Xu, et al. [33] analyzed vegetation indices derived from Landsat-7 ETM+ imagery for forest areas affected by Dendrolimus Punctatus Walker and obtained a kappa coefficient of 74.77% for samples with severe hazard classes. Consequently, it can be hypothesized that multispectral data and vegetation indices can achieve better results than RGB data when extracting pest-damaged forest areas from satellite images using a deep learning-based method.

Among the most obvious indicators for identifying pests and diseases of plants is the red-edge (680–780 nm). Variations in reflectance on the left side of the red-edge primarily reflect changes in the chlorophyll content of vegetation, whereas variations on the right side of the red-edge reflect changes in the leaf’s tissue structure and water content. Therefore, when the chlorophyll content of vegetation or the tissue structure within the leaves and the water content of vegetation change, the red-edge will shift, revealing the health status of the vegetation [34,35,36]. Existing studies have demonstrated that forest health monitoring models based on red-edge locations perform better than other models [37], with monitoring of vegetation stress in the red-edge and near-infrared bands is earlier compared to other bands [9,38,39,40,41,42]. Among optical satellites, Sentinel-2 is the only one with three bands in the red-edge area, providing information that is highly useful for monitoring the health of plants, and is favored by many forest-related researchers [43,44,45,46].

The attention mechanism has been extensively implemented in a variety of deep learning tasks, including natural language processing, image recognition, and speech recognition. Its essence is to selectively select input data so that the model focuses more on the important information in the data and ignores the irrelevant information. Multispectral remote sensing images have rich contextual semantic and spectral information, so it can be speculated that the addition of attention mechanism can enhance the segmentation of pest regions.

The complex surroundings as well as spectral and textural characteristics of pest regions in remotely sensed images pose a formidable challenge to their accurate extraction. This study proposes a new image segmentation method (called RSPR-UNet++) based on UNet++ [47] with an attention mechanism module for detecting forest damage induced by bark beetle and aspen leaf miner in Sentinel-2 images. There are blank spaces in research related to the problem of segmenting pest-infested forest areas, especially those connected to employing deep learning and multispectral satellite imagery. The main objective of this study are: (i) to exploit deep learning for improving forest-pest infestation area segmentation in multispectral satellite imagery; (ii) to illustrate the potential of multispectral data and vegetation indices for pest area segmentation by comparing with RGB images.

## 2. Study Area and Data

### 2.1. Study Area

**Figure 1:**
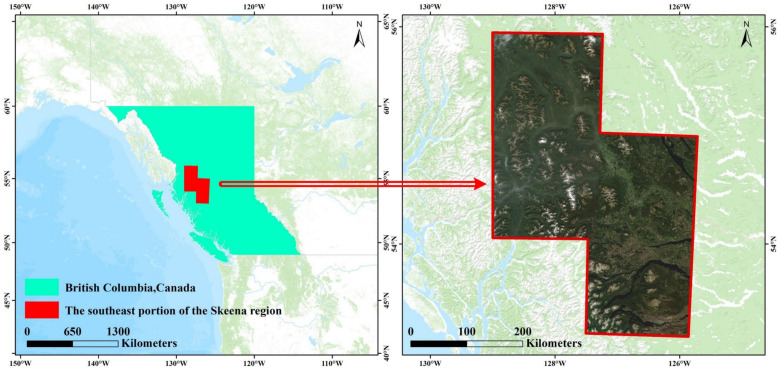
The study area’s location and an illustration of the Sentinel-2 images utilized for the experiment.

The study area is in the southeast portion of the Skeena region of BC, Canada (53°7′19″ N~55°39′12″ N, 125°52′3″ W~128°22′8″ W), covering an area of 48,224.16 km^2^. Since 2019, the Skeena region has experienced the greatest increase in mountain pine beetle and spruce beetle attacks in BC. The area damaged by aspen leaf miner in the region has nearly doubled since 2019, and the host species most affected by aspen leaf miner is trembling aspen, with a small percentage of other poplars also being damaged [48]. In the study area, the area affected by bark beetle is approximately 7200 km^2^ and the area affected by aspen leaf miner is approximately 4700 km^2^. Figure 1 depicts the study area’s geo-location together with an example map of the four Sentinel-2 scenes used in this study.

### 2.2. Sentinel-2 Data and Preprocessing

#### 2.2.1. Sentinel-2 Data

Sentinel-2 comprises two satellites, 2A and 2B, with one satellite having a 10 day revisit period and two complementary satellites having a 5 day revisit period. The Sentinel-2 data were downloaded from the U.S. Geological Survey’s data download site. We used Sentinel-2 data from 8 and 16 September 2020, to be as close as possible to the dates the Ministry of Forests, Lands, Natural Resource Operations and Rural Development (FLNRORD) conducted the 2020 aerial overview surveys (AOS) in the study area (late August to mid-September 2020) and to minimize cloud cover. Table 1 displays the Sentinel-2 band divisions and resolution for each band. The European Space Agency’s website provides additional information about the data characteristics of Sentinel-2 imagery.

#### 2.2.2. Data Preprocessing

The Sentinel-2 images obtained are multispectral Level 1C data, which are orthorectified and geometrically fine-corrected atmospheric-apparent reflectance products with no atmospheric correction. We utilized Sen2Cor to execute atmospheric correction on the downloaded Sentinel-2 1C level images, which resulted in 2A level images, with the B1 and B10 bands disappearing after correction.

As shown in Table 1, Sentinel-2 has three different resolutions for its bands: 10 m, 20 m, and 60 m. Many subsequent operations will not be possible if they are not unified to the same resolution. Consequently, following atmospheric correction, we resampled the images using the software Snap, resampling the bands with 20 m and 60 m resolutions to 10 m.

After resampling, we calculated NDWI, DWSI, NGRDI, RDI, GLI, NDRE2, PBI, NDVI, GNDVI, CIG, CVI, NDRE3, and DRS. Based on the common degree of vegetation indices in remote sensing image analysis and existing research results in pest research, 13 vegetation indices were calculated [49]. These vegetation indices were calculated using the bands of Sentinel-2 and the corresponding formulas; the calculation procedure is detailed in Table 2.

To avoid the effect of any extreme values that could be outliers, each vegetation index was ordered from smallest to largest and then linearly stretched to 0–255 (8-bit), with the value at 2.5% as the minimum and the value at 97.5% as the maximum. The original Sentinel-2 (12-bit) bands are directly scaled to 0–255. Each of these 13 vegetation index images was then added to the original image as a band.

### 2.3. Dataset

British Columbia, Canada, is largely forested, and forestry is a vital economic pillar for the province. As a result, the B.C. Ministry of FLNRORD conducts an annual AOS of the province’s forests in order to monitor their current state of health. The pest area labels for this study were determined using provincial fieldwork data from 2020.

**Figure 2:**
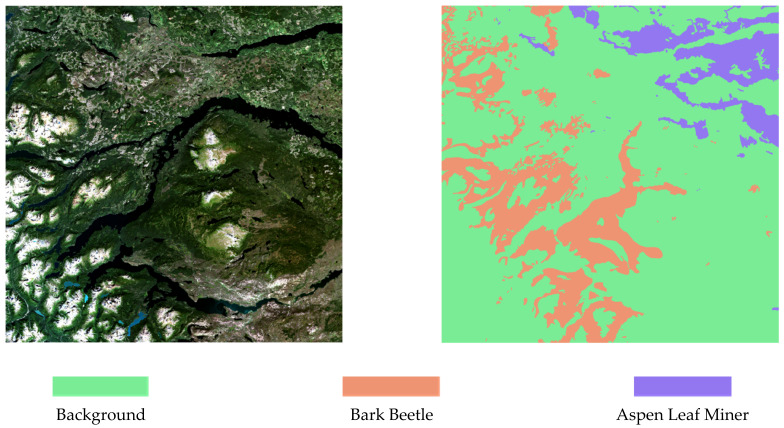
The raster label corresponding to the Sentinel-2 image.

Using Arcmap, the shapefile-formatted pest area labels are converted to raster form, which is the same size as the corresponding Sentinel-2 image. Using 1 of the 4 scenes of the Sentinel-2 image as an example, Figure 2 depicts the corresponding raster label of the image.

The remote sensing image and the corresponding label is cropped into several image blocks and then is inputted into the deep learning network to avoid memory overflow. In this study, the Sentinel-2 images and labels of the study area were cropped in a regular grid pattern to produce 4984 3D cubes and 4984 labels corresponding to 3D cubes. The length and width of the 3D cube are 256 pixels. The number of channels, i.e., the total number of original bands and vegetation indices, is 24. A total of 20% of the dataset was allocated to the test set, 64% to the training set, with 16% allocated to the validation set.

## 3. Methodology

### 3.1. Model Architecture

**Figure 3:**
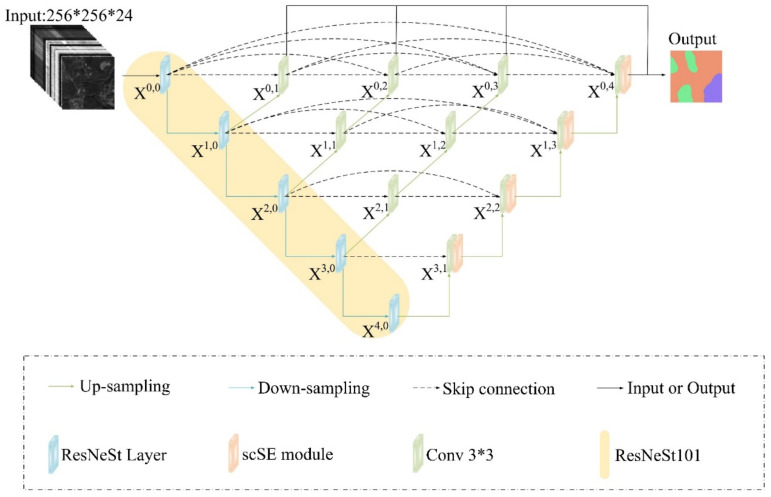
The structure of RSPR-UNet++.

Figure 3 shows the proposed model’s overall structure (called RSPR-UNet++). It consists of an encoder sub-network and a decoder sub-network. X^i,0^ (i = 0, 1, 2, 3, 4) stands for ResNeSt Layer and X^i,j^ (j≠0) stands for convolutional layer of 3*3 size. A 1*1 sized convolutional layer is added after each X^0,j^ (j≠0). Following the 1*1 convolutional layer is the sigmoid activation function. This design improves the gradient propagation by directly connecting X^0,j^ (j = 1, 2, 3) to the final output while supervising the output of the dense convolutional block of each branch. After experimental adjustment, the channels’ number of the feature-maps output from X^0,j^, X^1,j^, X^2,j^, X^3,j^ and X^4,j^ (j = 0, 1, 2, 3, 4) are set to 16, 32, 64, 128 and 256 (the original UNet++ is 32, 64, 128, 256 and 512) in turn.

**Figure 4:**
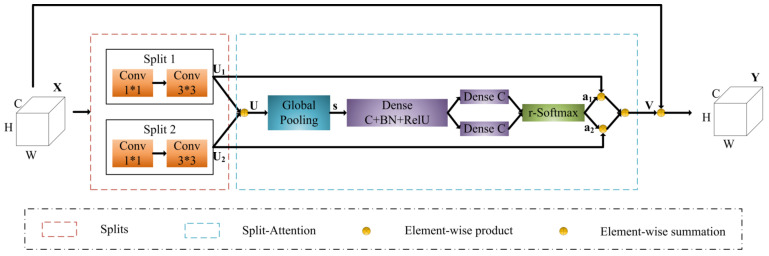
Split-Attention Block.

Based on the original UNet++ network, we use ResNeSt101 [50] as the feature extraction backbone network. ResNeSt introduces Split-Attention module while retaining the Residual Network’s structure (ResNet [51]), and stacks the Split-Attention module. The structure of the Split-Attention module in the model is shown in Figure 4. H, W, and C represent the height, width, and number of channels, respectively, of the input feature-map X. The module is a computational unit that consists of two parts: feature-map splits and split-attention operations. It divides the extracted feature-map into 2 feature-map splits, performs a series of transformations on each split, then fuses the weighted feature-maps after the transformations. Finally, as with the standard residual block, the final output feature-map Y is obtained by connecting with feature-map X and feature-map V using a shortcut connection. It realizes the information interaction between the feature-map splits, thus improving the model’s feature extraction capability. Using ResNeSt as the feature extraction backbone network, features with different weights can be obtained from different splits of feature-map, and richer feature information of the infested area can be extracted, making the segmentation results more accurate.

**Figure 5:**
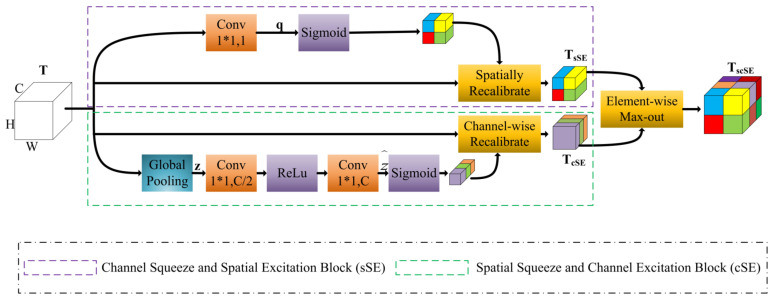
Spatial and Channel Squeeze and Excitation Block(scSE).

To further focus the model’s attention on the infested region and thus extract its more detailed features, we add the scSE [52] attention mechanism module at the end of each nested skip pathway in the decoding stage. Figure 5 depicts the specific structure of scSE, which consists of a combination of sSE module and cSE module. The scSE module calibrates and excites the spatial and channel features of the image in the application of pest region segmentation, reducing the influence of redundant features and effectively improving the model’s ability to automatically learn the image’s effective features, further enhancing the model’s segmentation accuracy.

Table 3 illustrates the characteristics of other common semantic segmentation models. RSPR-UNet++ combines attention mechanism and encoder–decoder structure, which is more advanced than them.

**Figure 6:**
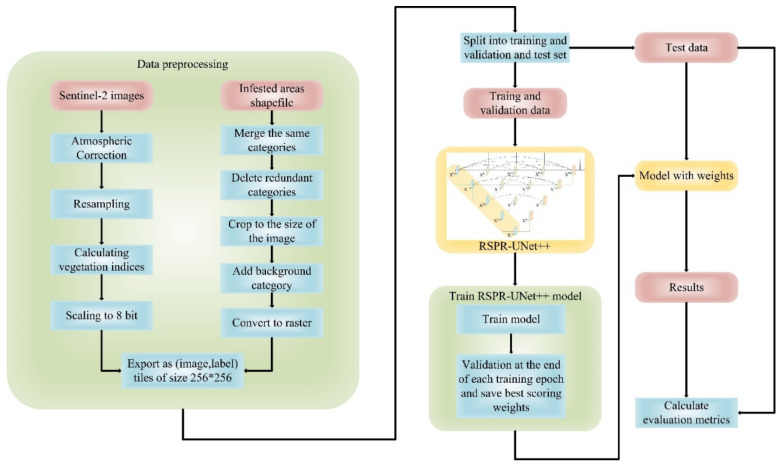
A flow chart of the process of creating the dataset together with the process of training, validating, and testing the model.

The flow of making the dataset as well as the process of training, validating, and testing the model are shown in Figure 6.

### 3.2. Loss Function

It is common in remote sensing images’ segmentation that the number of foreground pixels and background pixels differ significantly, i.e., there is a sample imbalance between the segmented object and the background. The Dice Loss function is more suitable for the case of sample imbalance, the specific formula is as follows:

(1) $$L_{\mathrm{dice}}=1-\frac{2\left|X⋂Y\right|}{\left|X\right|+\left|Y\right|}$$

where: X denotes the tensor of the true segmentation label of the image, and Y denotes the tensor of the image segmentation result predicted by the model. |X⋂Y| denotes the sum obtained by element-by-element summation for the result of the dot product of X and Y. |X| denotes the sum obtained by adding X element by element, and |Y| denotes the sum obtained by adding Y element by element.

In extreme cases, however, the Dice Loss function may cause the gradient values to be very high, which negatively impacts back propagation and renders model training unstable. Soft Cross Entropy Loss function does cross entropy calculation with the predicted values after label smoothing of the labeled values, which can improve the model’s generalization to some extent, the specific formula is as follows:

(2) $$L_{\mathrm{sce}}=-\overset{n}{\underset{i=1}{\sum }}p(x_{i})\log(q(x_{i}))$$

(3) $$x_{i\left(\mathrm{one}-\mathrm{hot}\right)}=\left\{\begin{array}{c} 1,x_{i}=\mathrm{target}\\ 0,x_{i}\neq \mathrm{target} \end{array}\right.$$

(4) $$p\left(x_{i}\right)=x_{i\left(\mathrm{one}-\mathrm{hot}\right)}*\left(1-\alpha \right)+\frac{\alpha }{K}$$

where: n denotes the number of pixel points, $x_{i}$ denotes a pixel point, and $q\left(x_{i}\right)$ denotes the probability that the model predicts $x_{i}$ to be the category in the labeling. K denotes the total number of categories in the segmentation, and K = 3 in this study. α denotes the smooth factor, and α = 0.1 in this study.

Consequently, the following equation combines the Dice Loss function with the Soft Cross Entropy Loss function as the experiment’s loss function:

(5) $$L=0.5{*L}_{\mathrm{dice}}+0.5{*L}_{\mathrm{sce}}$$

### 3.3. Model Training

The software environment used for the experiments is: Windows 10 Professional 64-bit system, Pytorch (1.8.0) framework, and Python (3.6). Hardware environment: 32 GB RAM; Intel(R) Core i7-10700, 2.90 GHz; NVIDIA GeForce RTX 2080Ti, 11 GB graphics memory.

The model training process is optimized using the AdamW optimizer, with the batchsize set to 6 and the weight decay set to 10^−3^, for a total of 300 training epochs. To improve the model’s convergence speed, we pretrain the ResNeSt101 network for migration learning using the ImageNet dataset. The gradient descent method may encounter local minima during training, and then the learning rate can be suddenly increased to “jump out”of the local minima and find the path to the global minima. Therefore, we use a cosine annealing strategy to adjust the learning rate. We let T_0 be the epoch of restart, T_mult be the factor after restart, and T_0 = T_0*T_mult after each restart. In this study, the initial T_0 = 2, T_mult = 2, the initial learning rate is 10^−4^, and the minimum learning rate is 10^−5^. Throughout the training process, the model with the highest mean Intersection-over-Union (mIoU) on the validation set is chosen as the final output model.

**Figure 7:**
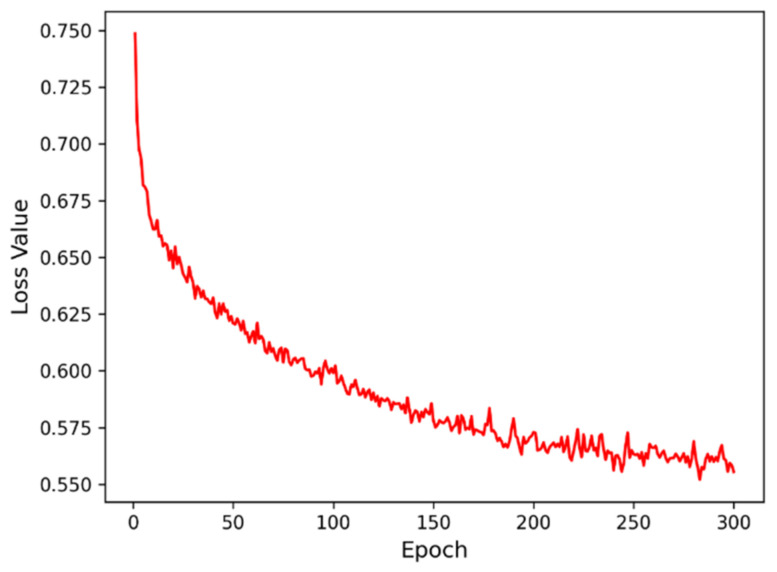
Loss value corresponding to different iterations.

The loss values during the training of the model are shown in Figure 7. It can be seen that the loss curve decreases rapidly at the beginning, then gradually converges to about 0.56 after about 225 iterations.

### 3.4. Evaluation Metrics

To demonstrate the efficacy of RSPR-UNet++ and compare each model’s performance on the test set, we quantitatively evaluated each model’s performance using accuracy, precision, recall, F1-score, Intersection-over-Union (IoU), mIoU, and frequency weighted Intersection-over-Union (FWIoU). These evaluation metrics are calculated as follows:

The accuracy is calculated as follows:

(6) $$\mathrm{accuracy}=\frac{\mathrm{TP}+\mathrm{TN}}{\mathrm{TP}+\mathrm{TN}+\mathrm{FP}+\mathrm{FN}}$$

where, TP (True Positive) denotes the number of true positive samples; FP (False Positive) denotes false positive samples; TN (True Negative) denotes true negative samples; and FN (False Negative) denotes false negative samples.

The precision and recall are calculated as follows:

(7) $$\mathrm{precision}=\frac{\mathrm{TP}}{\mathrm{TP}+\mathrm{FP}}$$

(8) $$\mathrm{recall}=\frac{\mathrm{TP}}{\mathrm{TP}+\mathrm{FN}}$$

The two metrics, accuracy and recall, sometimes appear contradictory and cannot be high at the same time, so they need to be considered together. The most common composite evaluation metric is the F1-score, which is the harmonic mean of the precision and recall, defined as:

(9) $$F1=2*\frac{\mathrm{Precision}*\mathrm{Recall}}{\mathrm{Precision}+\mathrm{Recall}}$$

The F1-score is determined by both precision and recall, when it is higher it indicates a better recognition result.

IoU is the ratio of intersection and union of the actual and predicted category samples, which is calculated as follows:

(10) $$\mathrm{IoU}=\frac{\mathrm{TP}}{\mathrm{TP}+\mathrm{FN}+\mathrm{FP}}$$

mIoU is the result of summing the IoU for each category and then averaging:

(11) $$\mathrm{mIoU}=\frac{\sum_{N}^{i}\mathrm{IoU}}{N}$$

where N is the number of categories.

FWIoU sets weights according to the frequency of occurrence of each category, and the weights are multiplied by the IoU of each category and summed:

(12) $$\mathrm{FWIoU}=\frac{\mathrm{TP}+\mathrm{FN}}{\mathrm{TP}+\mathrm{FP}+\mathrm{TN}+\mathrm{FN}}*\frac{\mathrm{TP}}{\mathrm{TP}+\mathrm{FP}+\mathrm{FN}}$$

### 3.5. Analyze the Impact of Different Data

To analyze the effects of RGB images, multispectral images, and vegetation indices, we compared and analyzed the results of training and testing of the proposed model using RGB, Sentinel-2’s original 11 bands, RGB plus 13 vegetation indices, and Sentinel-2’s original 11 bands plus 13 vegetation indices.

In addition, for the purpose of analyzing the influence of the red-edge and the vegetation indices derived from the red-edge, we added 8 vegetation indices related to the red-edge to the original vegetation indices. The new vegetation indices added are shown in Table 4. We compared and analyzed the results of training and testing of the proposed model using all 32 bands and the 18 bands of the 32 bands that are not related to the red edge (The 8 new vegetation indices, Vegetation Red Edge 1, Vegetation Red Edge 2, Vegetation Red Edge 3, NDRE2, NDRE3, and CVI were excluded.).

## 4. Results

### 4.1. Comparison of Predicted Results

#### 4.1.1. Comparison between Different Models

To verify RSPR-UNet++’s performance, in Table 5 we compare the commonly used semantic segmentation models listed in Table 3. All models were trained and tested using all 24 bands. It can be seen that RSPR-UNet++ has 5.98%, 4.41%, and 2.9% higher mIoU, FWIoU, and accuracy, respectively, compared with UNet; 4.73%, 3.75%, and 2.6% higher mIoU, FWIoU, and accuracy, respectively, compared with DeeplabV3+; mIoU, FWIoU, and accuracy, respectively, compared with FPN was 5.31%, 3.9%, and 2.59% higher; mIoU, FWIoU, and accuracy was 4.98%, 3.63%, and 2.4% higher, respectively, compared with PAN; mIoU, FWIoU, and accuracy was 4.64%, 3.44%, and 2.29% higher, respectively, compared with UNet++. A more accurate segmentation result was achieved by RSPR-UNet++ than other models.

To further investigate the impact of the ResNeSt and scSE modules on the proposed model. Table 5 compares the segmentation effect of RSPR-UNet++ with and without the scSE module. It can be seen that when the scSE module is removed, the mIoU falls by 0.99%, the FWIoU falls by 0.74%, and the accuracy falls by 0.5%. Compared to UNet++, RSPR-UNet++ with the scSE module removed improves mIoU by 3.65%, FWIoU by 2.2%, and accuracy by 1.79%. Both the ResNeSt and scSE modules have a positive effect on the model, as is evident.

**Figure 8:**
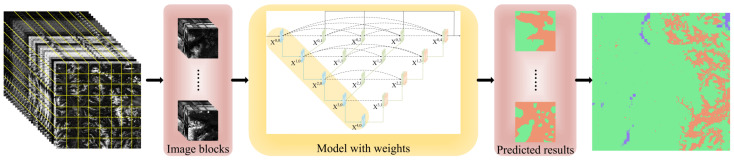
The process of the prediction of the entire Sentinel-2 image.

**Figure 9:**
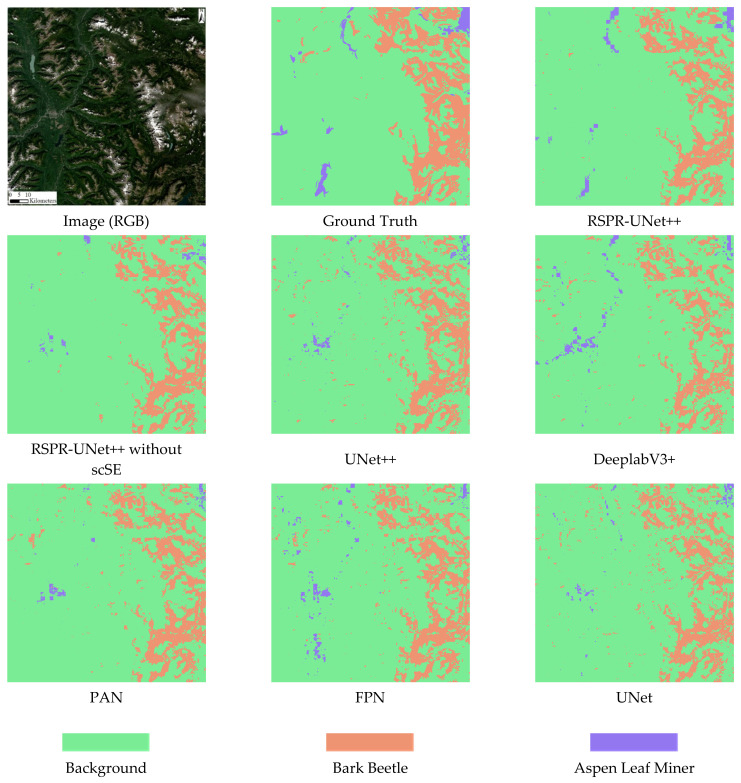
The segmentation effect of the whole Sentinel-2 image.

To compare the segmentation effects of RSPR-UNet++ and other models on the full-size Sentinel-2 image, we used a scene of Sentinel-2 image from the Skeena region with the imaging date of 28 August 2019. After preprocessing, it is cropped in regular grid fashion into a number of 3D cubes of size 256*256*24. Predictions are made for each cube, and then the predictions are stitched together sequentially to obtain the predicted result for the entire Sentinel-2 image. Figure 8 depicts the complete procedure. The segmentation effect of RSPR-UNet++ and other models on the full-size Sentinel-2 image are shown in Figure 9. Table 6 compares these models’ overall accuracy for the segmentation results of this Sentinel-2 image. In comparison to other models, RSPR-UNet++ has a superior segmentation effect.

#### 4.1.2. Comparison between RGB Data, Multispectral Data, and Vegetation Indices

**Figure 10:**
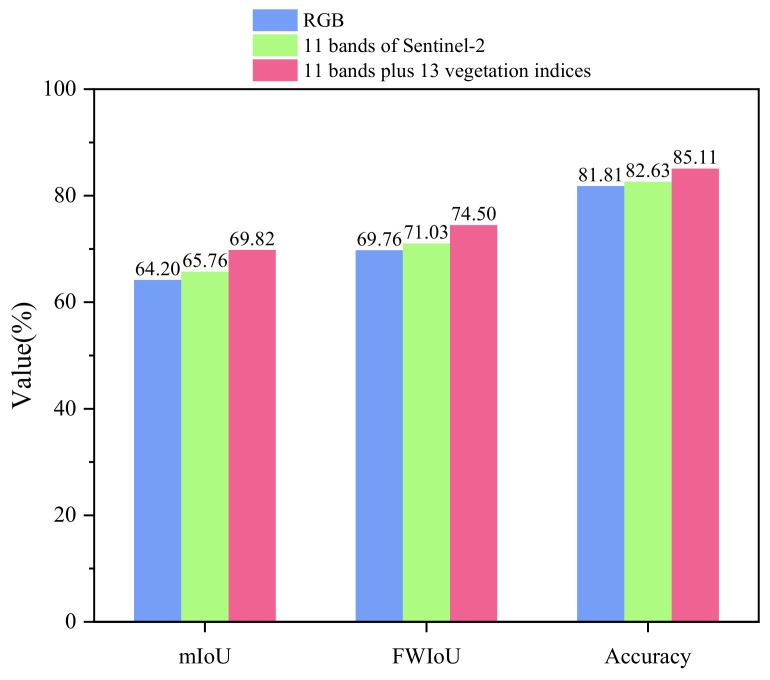
Comparison of the evaluation metrics after the two additions of the bands.

Figure 10 and Table 7 shows the experimental results of using RGB, Sentinel-2’s original 11 bands, and Sentinel-2’s original 11 bands plus 13 vegetation indices to train and test the proposed model. Compared with using RGB, when training with the original 11 bands of Sentinel-2, mIoU improved by 1.56%, FWIoU improved by 1.27%, and accuracy improved by 0.82%. It is clearly seen that, when training with all bands, mIoU improves by 4.06%, FWIoU improves by 3.47%, and accuracy improves by 2.48% compared to the original 11 bands of Sentinel-2.

To analyze the effects of the 13 vegetation indices and the remaining bands of Sentinel-2 except RGB, the results of training and testing by using RGB plus 13 vegetation indices are also compared in Table 7. Compared with the original 11 bands of Sentinel-2, the mIoU improved by 1.3%, the FWIoU improved by 0.98%, and the accuracy improved by 0.68% when using RGB plus 13 vegetation indices for training.

Table 7 also displays the experimental results of training and testing the proposed model using Sentinel-2’s original 11 bands plus 13 previous vegetation indices plus eight newly added vegetation indices related to red edge and the 18 bands of the 32 bands that are not related to red edge. After deleting the bands and vegetation indices associated with red edge from the 11 bands and the previous 13 vegetation indices, the mIoU decreased by 1.65%, the FWIoU decreased by 1.74%, and the accuracy decreased by 0.98%. In contrast, the mIoU decreased by 1.7%, the FWIoU decreased by 2.33%, and the accuracy decreased by 1.26% following the addition of 8 vegetation indices related to the red edge to the 11 bands and 13 vegetation indices.

### 4.2. Comparison of the Spectral Characteristics of Different Infestation Types

**Figure 11:**
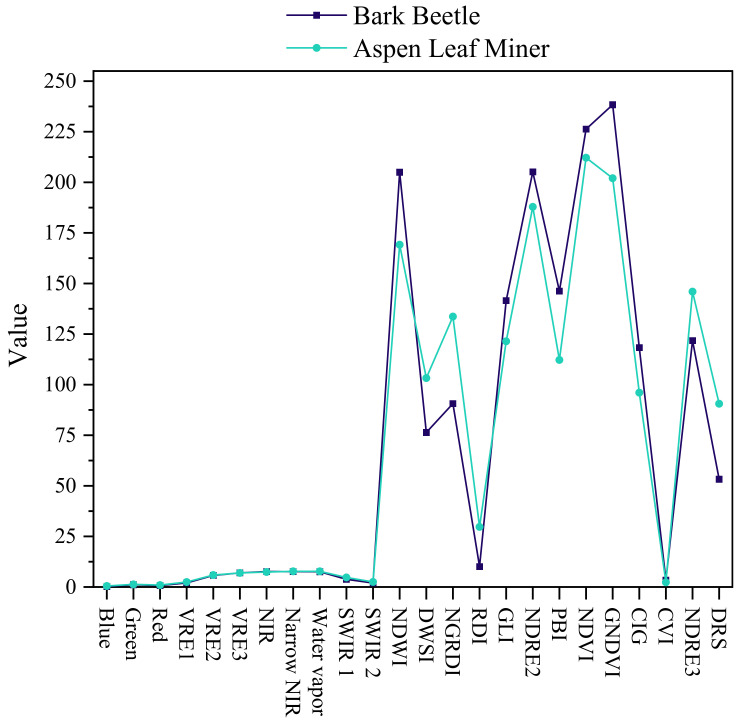
The spectral figure of bark beetle and aspen leaf miner. VRE represents Vegetation Red Edge.

We extracted the pixel values of each band for various types of pest-infested areas in the dataset and then averaged the pixel values of each band. As illustrated in Figure 11, the spectral figures of bark beetle and aspen leaf miner were acquired. The differences between bark beetle and aspen leaf miner on the original bands of Sentinel-2 are relatively small, whereas the differences between the vegetation indices, with the exception of CVI, are greater.

## 5. Discussion

We proposed a segmentation model based on Unet++ and named RSPR-UNet++ for the extraction from optimized Sentinel-2 images of bark beetle and aspen leaf miner infested regions. Compared with other common semantic segmentation models, RSPR-UNet++ introduces attention mechanism [57,58,59], and the model is more advanced in structure. For the optimization of Sentinel-2 images, we added 13 bands based on the formulae for 13 vegetation indices to the original bands, drawing on the existing research results in pest research and the common degree of vegetation indices in remote sensing image analysis. Previous study demonstrated the feasibility of employing satellite remote sensing technology to monitor forest pests [60,61,62,63,64]. The current research utilizing deep learning to detect forest damage employ RGB images. Multispectral satellite images and vegetation indices were not utilized in this field. We demonstrated the importance of vegetation indices and multispectral data to improve the segmentation effect with experiments. As far as we know, our research is the first exploitation of deep learning for forest-pest infestation area segmentation on multispectral satellite images, particularly those containing numerous vegetation indices.

As shown in Table 5, RSPR-UNet++ outperforms other models in terms of evaluation metrics such as accuracy, mIoU, and FWIoU for pest region segmentation. We speculate that the main reason is that RSPR-UNet++ employs ResNeSt for feature extraction and incorporates the scSE attention mechanism module. However, we were uncertain that both ResNeSt and scSE played a positive role in the segmentation effect, so we added ablation experiments to determine the effect of the scSE module and ResNeSt on RSPR-UNet++ independently. After RSPR-UNet++ eliminates the scSE module, virtually all evaluation metrics are reduced. We also added contrast experiments to explore the influence of cSE and sSE on the segmentation effect. According to Table 8, both cSE and sSE contribute positively to the segmentation effect. It suggests that the mechanism of recalibration and excitation of features from both spatial and channel dimensions by the scSE module enables the model to concentrate more on the features that are useful for detecting infested regions and suppress the useless features, thereby enhancing the segmentation effect. Comparing the evaluation metrics of RSPR-UNet++ without the scSE module to those of UNet++ reveals that nearly all of the former are superior to the latter, indicating that ResNeSt’s capacity to extract features is superior than the original UNet++’s encoder portion. The Split-Attention block within ResNeSt divides the input feature-map into feature-map splits, calculates weights for each feature-map split, and then combines the feature-map splits to produce a new feature-map. This procedure extracts additional characteristics of the infested area. ResNeSt and scSE both have the attention operation of assigning weights to features and combining new feature-maps, and they each positively contribute to RSPR-UNet++. It demonstrates that redundant features can have a negative impact on the model’s ability to extract pest regions from multispectral remote sensing images, which contain both rich contextual semantic and spectral information. The attention mechanism allows the model to concentrate more on useful characteristics, thereby improving the final results. Additionally, we adjusted the channels’ number of the feature-maps output from X^0,j^, X^1,j^, X^2,j^, X^3,j^ and X^4,j^ (j = 0, 1, 2, 3, 4) experimentally. As shown in Table 9, we attempted these three sets of parameters due to our hardware limitations.

As can be seen in Table 7, when training with the original 11 bands of Sentinel-2, nearly all evaluation metrics were enhanced compared to when RGB was used. It indicates that the other Sentinel-2 bands provide favorable characteristics for pest region segmentation. In addition, after adding 13 vegetation indices for training, nearly all evaluation metrics were further enhanced compared to Sentinel-2’s initial bands. Compared to the original 11 bands of Sentinel-2, it is indicated that the bands of Sentinel-2 plus 13 vegetation indices provide more useful features for pest area segmentation. Comparing the results of these three experiments, as depicted in Figure 10, reveals that the proposed model’s segmentation effect is improved more by adding 13 vegetation indices based on 11 Sentinel-2 bands than by adding the remaining Sentinel-2 bands based on RGB bands. We speculate that this is due to the fact that the 13 vegetation indices contain more characteristics than the native bands of Sentinel-2, excluding RGB. To further test this hypothesis, we employed a control variables approach, i.e., an experiment with RGB plus 13 vegetation indices. The results indicate that training with RGB plus 13 vegetation indices is superior to training with 11 Sentinel-2 bands. It indicates that adding 13 vegetation indices based on RGB improves the segmentation effect of the model more than adding the remaining bands of Sentinel-2 based on RGB. Our hypothesis was confirmed that vegetation indices can provide additional features such as spectral information about insect-infested and non-infested areas, thereby enhancing the model’s ability to differentiate between insect-infested and non-infested areas and enhancing segmentation performance. These four experiments also demonstrate that multispectral data and vegetation indices provide more effective features than RGB data for the task of segmenting insect-infested areas, where the internal chemical composition of trees is significantly altered, thereby enabling the model to segment the infested areas more precisely.

In addition, Table 7 shows that training the model with the data after removing the red-edge related bands and vegetation indices from Sentinel-2’s original 11 bands and previous 13 vegetation indices resulted in a decrease in the majority of evaluation metrics. This suggests that the red-edge bands and the vegetation indices derived from the red edges contain useful information for assessing forest health. Therefore, we hypothesize that increasing the red-edge related vegetation indices may improve the model’s precision. Consequently, we added eight red-edge-based vegetation indices to the Sentinel-2’s original 11 bands and previous 13 vegetation indices for training. Contrary to expectations, nearly all evaluation indices decreased, falling even lower than the results of training the model with the data after removing the red-edge-related bands and vegetation indices from Sentinel-2’s original 11 bands and previous 13 vegetation indices. We argue that this is due to the redundancy of information at this point, which has a negative impact on the model’s segmentation effect.

The above experiments illustrate that the nonlinear combination of the original bands is important. The information in the vegetation indices comes from the original bands. To some extent, all the information is contained in the original bands, and the vegetation indices make the information more obvious.

Although the model has similar results in segmenting bark beetle and aspen leaf miner in the test set, the segmentation results shown in Figure 9 show that the model detects bark beetle better than aspen leaf miner. We speculate that this is because the coverage area of bark beetle in the dataset is much larger than that of aspen leaf miner, which causes the model to be less generalizable to aspen leaf miner.

## 6. Conclusions

Accurate extraction of bark beetle and aspen leaf miner infected areas from remote sensing images is important for monitoring forest health and protecting forest ecosystems. In this study, a UNet++-based semantic segmentation model is proposed for segmenting bark beetle and aspen leaf miner infected regions in remote sensing images. In the encoder, we employ ResNeSt to improve the quality of the extracted features. To enhance the most important features for segmentation, the scSE module is introduced in the decoder. The experimental results demonstrated that the proposed model outperforms the state-of-the-art methods, such as UNet, DeeplabV3+, UNet++, etc. Moreover, to illustrate the potential of multispectral data and vegetation indices for pest area segmentation, we also implement the segmentation with RGB images, the results indicate that multispectral data and vegetation indices are more advantageous for pest area extraction since the vegetation indices can provide a wealth of information regarding the pest areas’ characteristics. Although this work focuses only on the forest pests of bark beetle and aspen leaf miner, our proposed method can also be extended for segmenting the other forest pest areas. In the future, investigating the optimal band and vegetation indices to further improve the segmentation performance will be interesting. The remote sensing data usually tend to suffer from various degradation, noise effects, or variabilities in the process of imaging [65]. Solving this problem and applying the solution to pest area extraction can also be a direction in future work.

## Figures and Tables

**Table 1:** The features of Sentinel-2 imagery.

Band	Band Name	Resolution (m)
B1	Coastal aerosol	60
B2	Blue	10
B3	Green	10
B4	Red	10
B5	Vegetation Red Edge 1	20
B6	Vegetation Red Edge 2	20
B7	Vegetation Red Edge 3	20
B8	NIR	10
B8A	Narrow NIR	20
B9	Water vapor	60
B10	SWIR-Cirrus	60
B11	SWIR 1	20
B12	SWIR 2	20

**Table 2:** Method for calculating vegetation indices.

Vegetation Indices	Calculation Method	Calculation Details in Sentinel-2
NDWI	$\frac{\left(\mathrm{Narrow}\;\mathrm{NIR}\right)-\left(\mathrm{SWIR}\;1\right)}{\left(\mathrm{Narrow}\;\mathrm{NIR}\right)+\left(\mathrm{SWIR}\;1\right)}$	$\frac{B8A-B11}{B8A+B11}$
DWSI	$\frac{\mathrm{NIR}+\mathrm{Green}}{\mathrm{Red}+\left(\mathrm{SWIR}\;1\right)}$	$\frac{B8+B3}{B4+B11}$
NGRDI	$\frac{\mathrm{Green}-\mathrm{Red}}{\mathrm{Green}+\mathrm{Red}}$	$\frac{B3-B4}{B3+B4}$
RDI	$\frac{\mathrm{SWIR}\;2}{\mathrm{Narrow}\;\mathrm{NIR}}$	$\frac{B12}{B8A}$
GLI	$\frac{2*\mathrm{Green}-\mathrm{Blue}-\mathrm{Red}}{2*\mathrm{Green}+\mathrm{Blue}+\mathrm{Red}}$	$\frac{2*B3-B2-B4}{2*B3+B2+B4}$
NDRE2	$\frac{\left(\mathrm{Vegetation}\;\mathrm{Red}\;\mathrm{Edge}\;3\right)-\left(\mathrm{Vegetation}\;\mathrm{Red}\;\mathrm{Edge}\;1\right)}{\left(\mathrm{Vegetation}\;\mathrm{Red}\;\mathrm{Edge}\;3\right)+\left(\mathrm{Vegetation}\;\mathrm{Red}\;\mathrm{Edge}\;1\right)}$	$\frac{B7-B5}{B7+B5}$
PBI	$\frac{\mathrm{NIR}}{\mathrm{Green}}$	$\frac{B8}{B3}$
NDVI	$\frac{\left(\mathrm{Narrow}\;\mathrm{NIR}\right)-\mathrm{Red}}{\left(\mathrm{Narrow}\;\mathrm{NIR}\right)+\mathrm{Red}}$	$\frac{B8A-B4}{B8A+B4}$
GNDVI	$\frac{\left(\mathrm{Narrow}\;\mathrm{NIR}\right)-\mathrm{Green}}{\left(\mathrm{Narrow}\;\mathrm{NIR}\right)+\mathrm{Green}}$	$\frac{B8A-B3}{B8A+B3}$
CIG	$\frac{\mathrm{Narrow}\;\mathrm{NIR}}{\mathrm{Green}}-1$	$\frac{B8A}{B3}-1$
CVI	$\frac{\left(\mathrm{Narrow}\;\mathrm{NIR}\right)*\left(\mathrm{Vegetation}\;\mathrm{Red}\;\mathrm{Edge}\;1\right)}{{\left(\mathrm{Green}\right)}^{2}}$	$\frac{B8A*B5}{{\left(B3\right)}^{2}}$
NDRE3	$\frac{\left(\mathrm{Narrow}\;\mathrm{NIR}\right)-\left(\mathrm{Vegetation}\;\mathrm{Red}\;\mathrm{Edge}\;3\right)}{\left(\mathrm{Narrow}\;\mathrm{NIR}\right)+\left(\mathrm{Vegetation}\;\mathrm{Red}\;\mathrm{Edge}\;3\right)}$	$\frac{B8A-B7}{B8A+B7}$
DRS	$\sqrt{{\left(\mathrm{Red}\right)}^{2}+{\left(\mathrm{SWIR}\;2\right)}^{2}}$	$\sqrt{{\left(B4\right)}^{2}+{\left(B12\right)}^{2}}$

**Table 3:** Other common semantic segmentation models and their characteristics.

Model	Characteristics	Reference
UNet	The architecture contains 2 paths (contraction path and symmetric expanding path). It is an end-to-end fully convolutional network (FCN).	[53]
DeeplabV3+	The spatial pyramid pooling module and the encoder–decoder structure were combined. The depthwise separable convolution was applied to both the Atrous Spatial Pyramid Pooling and decoder modules.	[54]
Feature Pyramid Networks (FPN)	Developed a top-down architecture with lateral connections for building high-level semantic feature maps at all scales.	[55]
Pyramid Attention Network (PAN)	Exploited the impact of global contextual information in semantic segmentation.	[56]
UNet++	The architecture is an encoder–decoder network where the encoder and decoder sub-networks are connected through a series of nested, dense skip pathways. It optimizes the topology of UNet and is an improved version of the UNet network structure.	[47]

**Table 4:** Method for calculating the 8 new vegetation indices related to red edge.

Vegetation Indices	Calculation Method	Calculation Details in Sentinel-2
ND790/670	$\frac{\left(\mathrm{Vegetation}\;\mathrm{Red}\;\mathrm{Edge}\;3\right)-\mathrm{Red}}{\left(\mathrm{Vegetation}\;\mathrm{Red}\;\mathrm{Edge}\;3\right)+\mathrm{Red}}$	$\frac{B7-B4}{B7+B4}$
NDVI690-710	$\frac{\mathrm{Water}\;\mathrm{vapor}-\left(\mathrm{Vegetation}\;\mathrm{Red}\;\mathrm{Edge}\;1\right)}{\mathrm{Water}\;\mathrm{vapor}+\left(\mathrm{Vegetation}\;\mathrm{Red}\;\mathrm{Edge}\;1\right)}$	$\frac{B9-B5}{B9+B5}$
NDRE	$\frac{\mathrm{NIR}-\left(\mathrm{Vegetation}\;\mathrm{Red}\;\mathrm{Edge}\;1\right)}{\mathrm{NIR}+\left(\mathrm{Vegetation}\;\mathrm{Red}\;\mathrm{Edge}\;1\right)}$	$\frac{B8-B5}{B8+B5}$
NDVI65	$\frac{\left(\mathrm{Vegetation}\;\mathrm{Red}\;\mathrm{Edge}\;2\right)-\left(\mathrm{Vegetation}\;\mathrm{Red}\;\mathrm{Edge}\;1\right)}{\left(\mathrm{Vegetation}\;\mathrm{Red}\;\mathrm{Edge}\;2\right)+\left(\mathrm{Vegetation}\;\mathrm{Red}\;\mathrm{Edge}\;1\right)}$	$\frac{B6-B5}{B6+B5}$
GNDVIhyper	$\frac{\left(\mathrm{Vegetation}\;\mathrm{Red}\;\mathrm{Edge}\;3\right)-\mathrm{Green}}{\left(\mathrm{Vegetation}\;\mathrm{Red}\;\mathrm{Edge}\;3\right)+\mathrm{Green}}$	$\frac{B7-B3}{B7+B3}$
RENDVI1	$\frac{\left(\mathrm{Vegetation}\;\mathrm{Red}\;\mathrm{Edge}\;1\right)-\mathrm{Red}}{\left(\mathrm{Vegetation}\;\mathrm{Red}\;\mathrm{Edge}\;1\right)+\mathrm{Red}}$	$\frac{B5-B4}{B5+B4}$
RENDVI2	$\frac{\left(\mathrm{Vegetation}\;\mathrm{Red}\;\mathrm{Edge}\;2\right)-\mathrm{Red}}{\left(\mathrm{Vegetation}\;\mathrm{Red}\;\mathrm{Edge}\;2\right)+\mathrm{Red}}$	$\frac{B6-B4}{B6+B4}$
RI	$\frac{\left(\mathrm{Vegetation}\;\mathrm{Red}\;\mathrm{Edge}\;1\right)-\mathrm{Green}}{\left(\mathrm{Vegetation}\;\mathrm{Red}\;\mathrm{Edge}\;1\right)+\mathrm{Green}}$	$\frac{B5-B3}{B5+B3}$

**Table 5:** The experimental outcomes of RSPR-UNet++ and other methods. BG represents the background, BB the bark beetle, and ALM the aspen leaf miner.

Model	Category	Precision (%)	Recall (%)	F1 (%)	IoU (%)	mIoU (%)	FWIoU (%)	Accuracy (%)
UNet	BG	85.44	88.63	87.00	77.00	63.84	70.09	82.21
	BB	76.64	69.74	73.03	57.52			
	ALM	74.03	71.26	72.62	57.01			
FPN	BG	86.33	87.93	87.12	77.18	64.51	70.60	82.52
	BB	75.09	74.12	74.60	59.49			
	ALM	75.08	70.11	72.51	56.87			
PAN	BG	86.39	88.34	87.36	77.55	64.84	70.87	82.71
	BB	75.00	73.05	74.01	58.75			
	ALM	76.15	71.21	73.59	58.22			
DeeplabV3+	BG	87.63	86.22	86.92	76.86	65.09	70.75	82.51
	BB	74.85	75.81	75.33	60.42			
	ALM	71.49	75.44	73.41	57.99			
UNet++	BG	86.81	87.81	87.31	77.47	65.18	71.06	82.82
	BB	75.34	74.71	75.03	60.03			
	ALM	75.00	71.93	73.44	58.02			
RSPR-UNet++ without scSE	BG	89.61	87.06	88.32	79.08	68.83	73.76	84.61
	BB	75.60	82.15	78.74	64.94			
	ALM	76.83	76.98	76.90	62.47			
RSPR-UNet++	BG	89.92	87.53	88.71	79.70	69.82	74.50	85.11
	BB	78.10	79.52	78.81	65.02			
	ALM	75.16	82.33	78.58	64.72			

**Table 6:** The overall accuracy for the segmentation results of the Sentinel-2 image in Figure 9.

Model	Accuracy (%)
UNet	86.79
FPN	86.31
PAN	87.40
DeeplabV3+	85.87
UNet++	87.49
RSPR-UNet++ without scSE	88.29
RSPR-UNet++	89.10

**Table 7:** Effect of different data on the segmentation result of infested areas using RSPR-UNet++.

Data	Category	Precision (%)	Recall (%)	F1 (%)	IoU (%)	mIoU (%)	FWIoU (%)	Accuracy (%)
RGB	BG	87.06	85.67	86.36	75.99	64.20	69.76	81.81
	BB	73.45	73.32	73.38	57.96			
	ALM	71.32	76.76	73.94	58.66			
11 bands	BG	88.96	84.98	86.93	76.87	65.76	71.03	82.63
	BB	70.04	81.40	75.29	60.38			
	ALM	76.43	73.65	75.02	60.02			
RGB plus 13 vegetation indices	BG	89.79	84.79	87.22	77.34	67.06	72.01	83.31
	BB	76.23	78.65	77.42	63.16			
	ALM	68.99	83.43	75.52	60.67			
11 bands plus 13 vegetation indices	BG	89.92	87.53	88.71	79.70	69.82	74.50	85.11
	BB	78.10	79.52	78.81	65.02			
	ALM	75.16	82.33	78.58	64.72			
8 bands plus 10 vegetation indices	BG	86.43	89.63	88.00	78.57	68.17	72.76	84.13
	BB	80.66	74.73	77.58	63.37			
	ALM	78.76	75.28	76.98	62.57			
11 bands plus 21 vegetation indices	BG	84.68	90.62	87.55	77.85	68.12	72.17	83.85
	BB	81.21	75.63	78.32	64.37			
	ALM	84.00	70.48	76.65	62.14			

**Table 8:** Effects of different attention modules.

Attention Module	Accuracy (%)
scSE	85.11
cSE	84.93
sSE	84.85
None	84.61

**Table 9:** The channels’ number of the feature-maps output.

Parameters	Accuracy (%)
16, 32, 64, 128 and 256	85.11
32, 64, 128, 256 and 512	85.03
64, 128, 256, 512 and 1024	83.24

## Data Availability

Data sharing is not applicable to this article.
